# Rubeola Complicated with Pneumonia

**Published:** 1865-02

**Authors:** P. J. Wardner

**Affiliations:** Springfield, Ill.


ARTICLE V.

### RUBEOLA COMPLICATED WITH PNEUMONIA.

BY P. J. WARDNER, M.D., of Springfield, Ill.

DR. DAVIS—*Dear Sir:*—In the fall of 1859, I witnessed the death of a promising young man with this serious malady, and was so much interested in the case that I gave much time and study to its treatment the following year. After which I resolved, should occasion offer, to make use of hyosciamus as a fundamental treatment; since which time I have never failed to control the disease with it to my perfect satisfaction. In my hands it has always acted as a specific. Usually, one prescription of it has completely overcome the rapid pulse, hot and dry skin, and pneumonic cough. I have long felt a desire to place the remedy before the profession, but have delayed for the purpose of ratifying my views by a thorough experience, and, having done so, I now offer this as a feeble contribution to medical science. The history of two cases may be advantageous to a clear understanding of its effect, and I herewith submit them:

CASE I. I was called, the 14th of Dec., 1859, to Mrs. N.; arrived at 6 P.M.; found the eruption had disappeared the previous day, and, thinking herself quite well, paid a visit to a neighboring house and returned in the evening, feeling some pain in the head, which increased until 4 in the morning, when sickness and vomiting supervened, and continued increasing up to my visit. I found the pulse beating 150 times per minute; skin hot and dry; lips parched, and tongue dry and red; and the distress at the stomach uncommonly severe, with a deathly sickness, almost incessant vomiting, and some cough. Ordered feet bathed in mustard water, and a cataplasm to epigastrum, to eat pounded ice, and take

| | | |
|---|---|---|
| ℞. | Ex. Hyosciamus, | gr. xxiv. |
| | Acetate Morphine, | gr. iij. |
| | Pulv. Acacia, | q.s. |
| | Ft. Pil. No. xij. | |

S.—Take one every two hours.

15*th*, 8 *A.M.*—Vomiting ceased at 11. Pulse 110; skin moist; cough increased, with expectoration tinged with blood. Continue pills; apply linen saturated in an infusion of hops and vinegar to chest.

6 *P.M.*—Cough decreasing; sputa clearer; pulse 98; skin moist; had eaten a ginger cookie at 5. Continue treatment.

16*th*, 9 *A.M.*—Patient sitting up; sputa clear; bowels moved at 7; pulse 88; skin normal; passed urine twice since last visit.

| | | |
|---|---|---|
| ℞. | Quinine Tannate, ------------------ | gr. xij. |
| | Ex. Hyosciami, ------------------- | gr. vj. |
| | Confection Roses, ------------------ | gr. s. |
| | Ft. Pil. No. vj. | |

Take one every six hours.

17*th*, 11 *A.M.*—Patient about the house. Continue pills one day. Discharged.

Case II. Capt. B. H. broke out with measles, on Sunday; eruption disappeared on Wednesday; on Thursday, was sent out with a scouting party in the rear of Memphis, Tenn.; returned on Saturday, and was placed in hospital and treated by Dr. —— for pneumonia following measles. The succeeding Thursday I was invited to visit the patient. Found a well-developed pneumonia, with restlessness; expectoration dark brown; a small wiry pulse; pallid countenance; a suppression of urine, and a dry, dirty tongue, with tenesmus. Advised,

| | | |
|---|---|---|
| ℞. | Ex. Hyosciamus, ------------------ | gr. xx. |
| | Sulph. Morph., -------------------- | gr. iij. |
| | Pulv. Columbo, -------------------- | q.s. |
| | Ft. Pil. No. xvj. | |

One every fours, alternating with an infusion of juniper berries and buchu.

Four days after, I saw the attending surgeon, who informed me that his patient had from my visit rapidly improved; and I ultimately learned that he was returned to duty the 11th day after my visit.

I have given these two cases, because they were the worst I have treated with the remedy. For young children, I place a small pill of the extract in a cup and add minute doses of antimony and morphine, or either, as the case may indicate, and add from four to six ounces of water. Then give a teaspoonful every two, three, or four hours, according to the circumstances and age of the patient.

SPRINGFIELD, Ill., Jan. 26th, 1865.

